# A new player in the field: Methylphenidate in post-traumatic stress disorder treatment

**DOI:** 10.1192/j.eurpsy.2021.1211

**Published:** 2021-08-13

**Authors:** R. López-Escribano, M.-J. Alvarez, J. Muñoz-Padrós, A. Marzán Gutierrez

**Affiliations:** Mental Health Department, Consorci Hospitalari de Vic, Vic, Spain

**Keywords:** post-traumatic stress disorder, methylphenidate, Dopamine

## Abstract

**Introduction:**

Currently available psychotherapies and psychotropic drugs for post-traumatic stress disorder (PTSD) are poorly effective in a substantial proportion of patients. Dopaminergic dysfunction plays a prominent role in the pathophysiology of PTSD: intrusions, avoidance symptoms, anhedonia and emotional numbing. Dopamine reuptake inhibitors can be studied as novel drugs in PTSD treatment.

**Objectives:**

Explore methylphenidate as a promising drug in PTSD treatment.

**Methods:**

Case report presentation based on the review of clinical notes and non-systematic review of the PTSD therapeutics state-of-the-art.

**Results:**

A 72-year-old Portuguese male, a veteran of the Angolan War, sought medical attention four years ago after the death of his brother, which had happened three years before the consultation. The clinical picture consisted of re-experiencing the war and the loss of his brother, flash-backs, nightmares, irritability, a fear of losing control, inner dialogues with occasional intra-psychic voices, emotional numbing with the impossibility of developing loving relationships with his relatives, feelings of unreality, an episode of dissociative fugue and complaints of episodic forgetfulness and time warp. He was diagnosed with PTSD with dissociative symptoms, based on DSM 5 clinical criteria. He was initially treated with SNRIs and risperidone, with little improvement. A year ago, he suffered a flare-up, with suicidal ideation. He was prescribed methylphenidate 36 mg, with progressive improvement, persisting mild PTSD residual symptoms.

**Conclusions:**

There is enough evidence of the dopamine involvement in PTSD, although research on dopaminergic drugs is scarce. Methylphenidate may be promising in the treatment of at least some individuals that haven’t responded to current psychological and medical interventions.

